# Tabla: A Proof-of-Concept Auscultatory Percussion Device for Low-Cost Pneumonia Detection †

**DOI:** 10.3390/s18082689

**Published:** 2018-08-16

**Authors:** Adam Rao, Jorge Ruiz, Chen Bao, Shuvo Roy

**Affiliations:** 1 Department of Bioengineering and Therapeutic Sciences, University of California, San Francisco, CA 94143, USA; shuvo.roy@ucsf.edu; 2 Department of Mechanical Engineering at University of California, Berkeley, CA 94720, USA; jorgeruiz@berkeley.edu (J.R.); chenbao@berkeley.edu (C.B.)

**Keywords:** acoustic, sensors, actuators, physiologic sensing, transfer function, spectrogram

## Abstract

Pneumonia causes the deaths of over a million people worldwide each year, with most occurring in countries with limited access to expensive but effective diagnostic methods, e.g., chest X-rays. Physical examination, the other major established method of diagnosis, suffers from several drawbacks, most notably low accuracy and high interobserver error. We sought to address this diagnostic gap by developing a proof-of-concept non-invasive device to identify the accumulation of fluid in the lungs (consolidation) characteristic of pneumonia. This device, named Tabla after the percussive instrument of the same name, utilizes the technique of auscultatory percussion; a percussive input sound is sent through the chest and recorded with a digital stethoscope for analysis. Tabla analyzes differences in sound transmission through the chest at audible frequencies as a marker for lung consolidation. This paper presents preliminary data from five pneumonia patients and eight healthy subjects. We demonstrate 92.3% accuracy in distinguishing between healthy subjects and patients with pneumonia after data analysis with a K-nearest neighbors algorithm. This prototype device is low cost and simple to implement and may offer a rapid and inexpensive method for pneumonia diagnosis appropriate for general use and in areas with limited medical infrastructure.

## 1. Introduction

Lung infections cause approximately 3.5 million deaths each year, and pneumonia, one type of lung infection, caused the deaths of almost a million children worldwide in 2015 [1,2]. Thus, pneumonia was the leading cause of death among children under the age of 5 in that period, with almost half of these deaths occurring in South Asia and sub-Saharan Africa [2]. Notably, these are areas with high rates of poverty and limited access to advanced medical infrastructure. As a result, the development of new, affordable diagnosic methods for pneumonia is a priority; UNICEF has reported that the development of such methods is necessary to reduce mortality among these vulnerable populations [3].

### 1.1. Current Clinical Technologies

The most common diagnostic techniques for pneumonia include physical exam and chest radiography. While other techniques such as ultrasound and Computed Tomography (CT) can be used for diagnosis, they are less common in clinical practice [4,5]. The chest X-ray, which detects the accumulation of protein-rich inflammatory fluid (exudate) in the lungs, is the current clinical standard for diagnosis of pneumonia. Despite this, the interpretation of chest X-rays is subject to interobserver disagreement between radiologists: the overall disagreement rate on the presence or absence of pneumonia between two staff radiologists can be as high as 20% [6]. An ideal diagnostic tool will have both high sensitivity (defined in this case as correct identification of disease in patients with pneumonia) and high specificity (no false positives in patients without pneumonia). The sensitivity and specificity of chest X-rays have been reported as 74% and 84%, respectively, falling in a range considered fair-to-good [7].

While chest radiography is effective in diagnosing pneumonia, it is inaccessible to certain patient populations due to prohibitive financial cost and/or a lack of access to the necessary infrastructure. For these populations, diagnosis via pulmonary physical examination—a technique all physicicans are trained to administer—is an accessible, convenient, and low-cost alternative [8]. Controlled studies have shown that auscultatory percussion, the physical examination technique in which the physician taps on the patient’s chest and listens to the patient’s back with a stethoscope, is a method capable of detecting various presentations of pneumonia [9]. Unfortunately, compared to a chest X-ray, the sensitivity (58%) and specificity (67%) of physical examination are fairly low [10]. This is due in part to differences in the interpretation of the findings; rates of interobserver agreement between two internists and a pulmonologist for diagnosing pneumonia via physical exam ranged from 60–72% in one study [10]. The undesirably high interobserver error of physical exams for diagnosing pneumonia has inspired researchers to seek new low-cost methods that offer improved sensitivity and specificity by quantifying physical findings.

### 1.2. Previous Studies on Acoustic Analysis of the Chest

Several studies have explored acoustic changes in the chest that result from diseases of the lung using electronically recorded output signals, both with and without the use of automated input signals. Electronic recording provides a more accurate and sensitive analytical platform than the human ear. Automated input signals provided by an electronic transducer are desirable because they can reduce variation while increasing reproducibility and interrater agreement.

A previous study investigating lung acoustics used an electromagnetic shaker placed on the sternum to provide automated input and measured vibrations of the back using a laser Doppler vibrometer [11]. This system was used to detect pneumothorax, a condition in which air accumulates in the lungs collapsing the lung tissue. The results were promising, but the input device was heavy (>2 kg) and would likely prove difficult to incorporate into the standard physical exam. For patients in the hospital who are mechanically ventilated or intubated, a speaker can be placed in the chest through the throat via endotracheal tube [12]. While this invasive method is reasonable for use with intubated patients, it is not appropriate for use in other populations.

Automated methods of sound input have not yet been applied for pneumonia diagnosis in the clinic, although the approach has shown promising results in several studies. One study reported a sensitivity of 90% and a specificity of 80% when multisensor recording of breath sounds was used for pneumonia diagnosis, values comparable to chest radiography [13]. Although these results are encouraging, they are influenced by case-mix and severity of pneumonia. Furthermore, the device used in that study is not compact, incorporating 40 sensors and a low-suction vacuum unit, components that limit its portability and increase its cost. Another study of a more compact device, which recorded breath sounds with a single microphone, reported a sensitivity of 72% and specificity of 82% for pneumonia diagnosis, with a classification accuracy by a neural network classifier of 77.6% [14]. In addition to breath sounds, speech can also be analyzed to detect pneumonia. In one study, subjects hummed a low vowel sound, which was recorded via an electronic stethoscope and analyzed to detect the presence of pneumonia [15]. Because it relies on a continuous hum, this approach would likely be very difficult to use when working with infants or non-vocal patients.

### 1.3. Acoustic Properties of the Lungs

Pulmonary physical exam maneuvers test the acoustics of the respiratory system, in particular the resonance, absorption, and transmission of sound traveling through the chest cavity [16].

Resonance refers to the natural frequency at which a structure will continue to vibrate when excited by an impulsive force; for the chest, this characteristic depends on the size of the thorax. For men, the resonant frequency of the chest is around 125 Hz; for women, it is slightly higher, at 150–175 Hz; and still higher for children, at 300–400 Hz [16]. The transfer function for the chest cavity describes its frequency response to percussion; it should have peaks at these resonant frequencies.

Absorption of sound occurs when energy is dissipated in a medium. When all frequencies are equally affected, absorption will increase the deeper the sound travels into the medium. Analysis of sound transmission suggests that the chest acts as a low-pass filter that absorbs frequencies above 1000 Hz as sound travels through the chest cavity [15,16,17].

The transmission of sound waves is altered at interfaces between different media in the chest cavity; in particular, the semi-rigid chest wall, pleural spaces (which can collect an excess of fluid in disease), and lung tissue, which is typically approximated as a homogeneous mixture of gas and tissue [18]. The fraction of sound intensity transferred at an interface can range from approximately zero (most of the sound being reflected at the interface) to one when the two bordering media have similar composition, and therefore similar acoustic impedance.

### 1.4. Pathophysiology and Physical Exam Findings in Pneumonia

**Figure 1:**
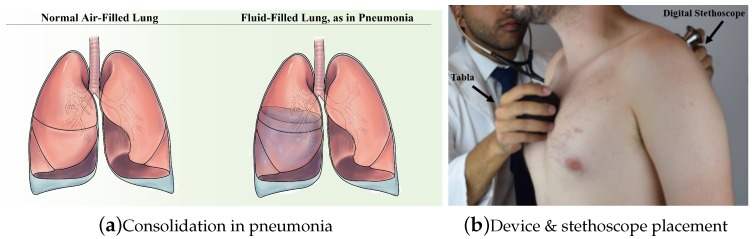
(**a**) a lung with consolidation characteristic of pneumonia and a healthy lung. Pneumonia leads to the accumulation of protein-rich fluid (exudate) in the lungs. The presence of exudate can be detected using different techniques, e.g., chest X-rays and acoustic analysis; (**b**) the Tabla is placed on the sternum and the stethoscope is moved to different quadrants of the lungs to record audio data, which is transmitted via Bluetooth to a smartphone (not shown). Figure from [22].

The lungs come into constant contact with particulate matter and microbes from the upper respiratory tract during normal breathing. Pneumonia develops when a microbe reaches the lungs and is not cleared by the immune system, either due to a defect in host immunity, exposure to a particularly virulent microbe, or microaspiration [19]. Infection of the lungs leads to inflammation and the accumulation of exudate (see Figure 1a). The accumulation of fluid at the site of infection provides the physiological basis for physical exam findings of pneumonia: egophony, dullness to percussion, increased tactile fremitus, changes in breath sounds and crackles, among others [20]. Explanation of each of the pulmonary physical exam maneuvers is outside the scope of this article, but a detailed summary can be found in [21]. Our device relies specifically on automation of the physical exam procedure of auscultatory percussion.

Percussion involves the physician tapping on the sternum and specific areas of the back and listening to the resulting sounds; experience allows the physician to determine if these sounds correspond to the presence of healthy or abnormal tissue. In a normal lung, much of the sound is reflected at the interface between the soft lung tissue and the semi-rigid chest wall, due to the large impedance mismatch. This leads to a “clear long lasting sound described as resonant” [23]. In the case of pneumonia, exudate surrounds the soft lung tissue and the acoustic impedance mismatch between the chest wall and the consolidation is minimal. Vibrations on the surface rapidly propagate to the exudate, leading to a “sound of low amplitude and short duration described as dull” [23]. The principle of operation for our device is the same as for auscultatory percussion, in which a physician applies a sound input by tapping the sternum with one hand listening with a stethoscope held on the back with the other hand [9]. The physician assesses the acoustic properties of the sound that travels through the chest.

## 2. Materials and Methods

In this section, we describe the design of the Tabla device from basic principles. Our design choices are intended to comply with the guidelines for a practical diagnostic tool for pneumonia set forth by United Nations Children’s Fund (UNICEF) [3], summarized in Table 1.

### 2.1. Approach and Statement of Contributions

The objective of this paper is to propose a quantitative alternative to the pulmonary physical exam that provides improved sensitivity and specificity, in addition to reducing interobserver error. The contributions of this paper are threefold. First, we propose diagnosing pneumonia by measuring the acoustics of the lungs with automated input and output devices. This has the dual advantages of improving the quality of the test (sensitivity and specificity) and clarity of the results (reducing interobserver error). While previous work has focused on fully automated tests for pneumothorax [11,12] or partially automated tests for pneumonia [13,15], this approach is novel because it automates both input and output for detecting pneumonia and provides quantitative results. Second, we provide a small, easy-to-use prototype which follows design criteria set forth by UNICEF [3]. The device consists of an automated input unit which we constructed together with a commercial, off-the-shelf electronic stethoscope (see Figure 1b). When a button is pressed, the input unit generates a ‘chirp’ signal and the vibrations on the back are recorded. A spectrogram of the recording is used to estimate the acoustic frequency response of the chest, which gives quantitative information useful for diagnosing pneumonia. Finally, we present initial clinical data with five patients collected under Institutional Review Board (IRB) study number 15-16814 at University of California, San Francisco Medical Center, and a proposed quantitative method of discrimination between healthy and consolidated lung tissue based on Mel-Frequency Cepstral Coefficients (MFCC) and spectral centroid features of the frequency response of the chest.

### 2.2. Principle of Operation

We chose to focus on an acoustic approach because the sensitivity and specificity of such systems in diagnosing pneumonia has been demonstrated (as mentioned in Section 1). This choice addresses the two primary design criteria of accuracy and reliability, and has the additional benefit of removing the radiation exposure inherent in methods such as the chest X-ray.

Much like the physician’s hand and stethoscope in auscultatory percussion, our device consists of two components: a sound source (actuator) and a sensor (stethoscope) (Figure 1b). The actuator uses a surface exciter to send a sound wave with a frequency sweep from 50 to 1000 Hz into the chest. The stethoscope is placed on the back and records the sound waves transmitted through the chest. All patient data is collected and stored on a Health Insurance Portability and Accountability Act (HIPAA)-compliant server for later analysis with Matlab (R2017b, MathWorks, Natick, MA, USA).

Previous work suggests that breath sounds in the frequency range 150–250 Hz provide vital information for pneumonia diagnosis (as discussed in Section 1). Characterization of the cutoff frequency of normal lungs suggests that frequencies above 1000 Hz do not contain useful data [15,17,24]. Furthermore, fixed-input studies of pneumothorax suggest that an upper limit of 400 Hz is necessary to keep the signal-to-noise ratio (SNR) in an appropriate range [12]. We expanded this range to 1000 Hz for our human subject recordings without undesirable effects on SNR.

### 2.3. Actuator

The choice of actuator was based on several considerations: its size, which affects the portability, cost and comfort of the device; and its intensity at low frequencies as well as its frequency range, both of which affect the ease of use and reliability of the device.

We considered several types of actuator, including piezoelectric transducers, push/pull solenoids, speakers, and surface exciters. The frequency range and power of each of the potential inputs was measured by its application to two different body parts: the knee, which is solid and provides a dull response; and the stomach, which is hollow and provides a resonant response. A comparison of each actuator type’s performance on each of the UNICEF criteria summarized in Table 1 is provided in Table 2.

Piezoelectric transducers are compact and inexpensive, both desirable features; however, they did not produce a signal of measurable strength. Solenoids, in contrast, provided strong low-frequency signals, but are bulky and have high power needs, contributing to shorter device battery life. Bluetooth speakers have been developed to meet consumer market demand for low weight, small size, and long battery life. Despite being rated for use in the 20 Hz–20 kHz range, many handheld speakers cannot consistently generate frequencies <100 Hz, which makes them inappropriate for use in this device. Surface exciters are a type of sound generator that are essentially speakers with no frame or cone; upon receiving an input signal, they vibrate an adjacent surface [25]. They are capable of consistently producing measurable sounds at frequencies as low as 50 Hz and are small enough to be incorporated in handheld devices. Based on these desirable characteristics, we chose to use a surface exciter (DAEX30 HESF-4, Dayton Audio, Springboro, OH, USA) in the design of the Tabla device.

**Figure 2:**
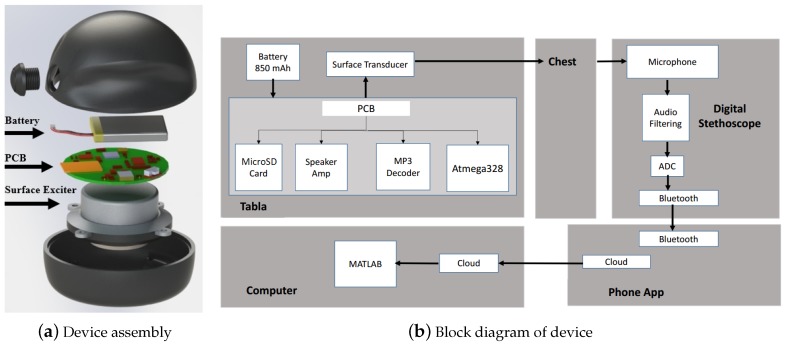
(**a**) the components of the device from top to bottom: 3D-printed plastic enclosure, button to start signal generation, Lithium polymer battery, printed circuit board (PCB), surface exciter; (**b**) the device prototype has four major components. Tabla transmits automated input into the chest and the Eko Core stethoscope (Eko Devices, Berkeley, CA) records output from the subject’s back. The sound files are transmitted from the stethoscope via Bluetooth and recorded using a smartphone app; the data are then stored on the cloud, as described in Section 2.5. Figure from [22].

As shown in the block labeled “Tabla” in Figure 2b, the actuator is driven by an amplifier circuit that uses MP3 data stored on a microSD card and processed by a VS1053B MP3 decoder chip (VLSI Solution, Tampere, Finland) controlled by an ATmega328p microprocessor (Atmel, San Jose, CA, USA). The device has a handheld enclosure that contains the battery, surface exciter, and printed circuit board (PCB). This enclosure has a shell consisting of manufacturing-grade resins with the properties of injection-molded thermoplastics (Carbon 3D, Redwood City, CA, USA), designed using SolidWorks 3D CAD software (Dassault Systems, Waltham, MA, USA) and 3D printed via stereolithography with Carbon 3D (Carbon 3D, Redwood City, CA, USA).

### 2.4. Sensors

Like the choice of actuator, the choice of sensor was influenced by the UNICEF guidelines for devices of this type. There are a number of small digital stethoscopes on the market which provide high-quality data at a reasonable cost [26,27,28]. We compared different options and found that the Eko Core [27] stethoscope offers several features appropriate for our application. Notably, the Eko Core has a similar form to the traditional stethoscope and can function as a regular stethoscope if the microphone is turned off. In our initial product feasibility studies, we found familiarity of both form factor and procedure are important in increasing comfort with, and thus the adoption of, new devices among medical practitioners. Although the Eko Core was chosen for this study, other digital stethoscopes, such as the 3M Littmann (3M, Maplewood, MN, USA) and Thinklabs One (Thinklabs, Centennial, CO, USA) should offer similar performance.

Wireless Bluetooth pairing of the stethoscope with a smartphone enables cloud storage of recorded data, facilitating its further use and analysis in telemedicine applications. In this proof-of-concept study, an iPhone 7 (Apple Inc., Cupertino, CA, USA) was used for data collection; data were recorded locally and stored in the cloud (Figure 2b).

### 2.5. Signal

Considerations that influenced the choice of input signal were the ability to streamline information processing, which affects the ease of interpretation and training for users; and the ability to quickly assess the patient, which affects the time required for diagnosis.

There are a number of well-established approaches to system identification. The impulse response is a very brief, sharp input that most closely mimics the tapping of physical exams. Its main benefits are speed and uniform power distribution across frequency bands. However, because of its short duration, it requires a large signal to compensate for noise [29] and is very demanding of the actuator. The slow sine sweep uses slowly changing frequencies, which allow the system to settle to a steady state. The main benefit is a very high signal to noise ratio, but it takes several minutes to run the test. We chose to use a ‘chirp’, a sinusoidal signal characterized by rapid frequency change. This provides a balance between the slow sine sweep and impulse response, and has the added benefit that it is easy to automatically extract the transfer function.

**Figure 3:**
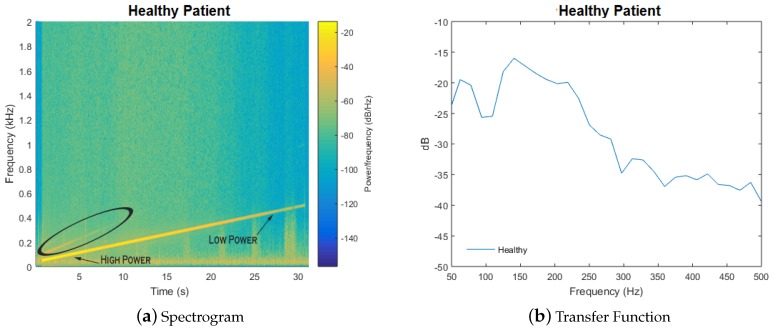
(**a**) spectrogram of a chirp signal applied to the chest of a healthy subject. Resonance is represented by a bright spot, as indicated by the arrow on the left side of the figure. Along the line of the chirp, a comparison to a less-bright spot is indicated on the right; harmonics are indicated by the non-parallel lines (circled) above the chirp; (**b**) plotting power values along the line of the chirp yields a plot of the transfer function. Figure from [22].

We analyzed the data with a spectrogram, which is the result of performing successive discrete Fourier transforms within an overlapping time window to estimate signal power as a function of frequency and time. An illustration of the spectrogram is shown in Figure 3a. The transfer function is extracted by using power measurements along the line, which corresponds to the input chirp signal, shown in Figure 3b. The full test takes only 15 s.

### 2.6. Signal Processing

The fixed input chirp signal lasted for 14 s. Periods of silence and background noise were cropped out of the signal to ensure accurate feature extraction. A cross-fade (500 ms fade in and fade out) was also performed to increase Discrete Fourier Transform (DFT) accuracy [30].

MFCCs and spectral centroid values were calculated from this pre-processed data set. MFCCs are a set of 10 coefficients that succinctly represent a frequency spectrum, commonly used to distinguish differences between spectra for, e.g., speech recognition [31]. In our case, we utilize the MFCCs to provide a fingerprint for the spectra of healthy subjects and compare these fingerprints to those of pneumonia patients. The spectral centroid is also used to characterize the spectrum, in this case based on which frequencies are most prominent, and has been used to help distinguish between different musical instruments [32]. For each patient, we averaged the MFCC and spectral centroid values across three trials and six lung regions. The open source Essentia library was used to calculate the MFCC and centroid values. The MFCC coefficients and spectral centroid value resulted in a set of 11 features to compare for each patient.

### 2.7. Classification

The K-nearest neighbors (KNN) algorithm was used to classify the feature set for each subject into the healthy or pneumonia group. KNN works by choosing the majority class of the K most comparable subjects. In this case, subjects are compared using Euclidean distance between the MFCC and centroid features. For this analysis, K values of 1, 3, 5, and 7 were compared to maximize algorithm performance.

## 3. Results and Discussion

In this section, we present and discuss several experiments performed with Tabla. In Section 3.1, we compare the measured acoustic properties of wet and dry sponges, which share a similar density with the lungs [33]. In Section 3.2, we present results on the acoustic properties of the knee, which is composed of solid tissue, versus the stomach, which is hollow. Finally, in Section 3.3, we present preliminary clinical data collected at University of California, San Francisco (UCSF) Medical Center from five pneumonia patients and eight healthy subjects.

### 3.1. Water Filled Sponge vs. Air Filled Sponge

We use sponges as a bench-top model for pneumonia since they mimic the texture and density of the lungs [33]. Lung density normally ranges between 0.2–0.5 gm/mL [16]. A hydrophilated grouting sponge QEP 70005Q-6D (QEP, Boca Raton, FL, USA) was chosen for density comparable to human lung. When the sponge is dry, it is slightly lower in density than that of the lungs at 0.05 gm/mL. Our device was placed on one side of the sponge with the stethoscope placed on the other side. Next, the sponge was filled with water until an effective density of 0.6 gm/mL similar to the density of consolidation was obtained. A set of 10 measurements was taken for both the dry and wet sponge, and a mean was calculated.

**Figure 4:**
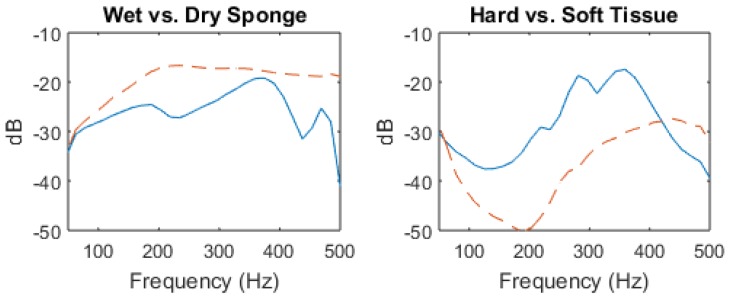
The frequency spectrum from 0–500 Hz for sound signals recorded for the wet (dotted line) vs. dry (solid line) sponge, and for the stomach (soft tissue, solid line) vs. knee (hard tissue, dotted line). Lines represent mean values from 10 consecutive measurements. The sponge experiment findings are consistent with physical predictions that accumulation of water, which reduces acoustic mismatch, leads to improved transmission of higher frequencies. The response for the knee is similar to the wet sponge and appears to behave as a high-pass filter; however, the effect is less pronounced and begins to attenuate above 450 Hz. Figure from [22].

The results are shown in Figure 4. For the dry sponge, we observe a resonant peak at 180 Hz and its first harmonic at 360 Hz, with higher frequencies being attenuated. The wet sponge behaves like a high-pass filter with decreased absorption of sound waves above 200 Hz; note that higher frequencies are less attenuated than in the dry sponge frequency response. These model findings are consistent with physical predictions that accumulation of water (which reduces acoustic mismatch) leads to improved transmission of higher frequencies. Specifically, the introduction of water led to an increase of transmitted sound intensity at 500 Hz of approximately 40 dB.

### 3.2. Solid Tissue vs. Air-Filled Cavity Response

Solid areas of the body, such as bone, typically result in “dull” findings upon percussion [34]. Based on percussion data, the knee was chosen as a model for consolidation. The device was placed on the anterior portion of the knee, and the stethoscope placed on the posterior portion. Tympanic sound is found over air filled cavities, and represents the opposite extreme from ”dull” findings. Typical tympanic sound is found over the stomach, where percussion is commonly utilized to determine liver span [34]. The device was placed on the stomach and the stethoscope placed on the back; the findings can be seen in Figure 4. Note that, due to the increased thickness of the stomach, as well as the improved sound conduction of bone, the volume of the input signal was adjusted to compensate. Since we are concerned with frequency patterns rather than amplitude, this change should not interfere with our results.

The results are similar to the model system, with resonant peaks notable in the frequency response of the air filled stomach, and high-pass filter characteristics in the frequency response of the knee. For the stomach, resonant peaks can be seen between 200–400 Hz. However, frequencies above 400 Hz are filtered out. The response for the knee is similar to the wet sponge and appears to behave as a high-pass filter; however, the effect is less pronounced and begins to attenuate above 450 Hz. The measured increase in transmitted sound intensity of approximately 10 dB at 500 Hz is also less dramatic than in the sponge experiment, indicating that the difference between the stomach and the knee is less pronounced than the wet and dry sponges.

### 3.3. Clinical Proof of Concept

Under an IRB-approved study at the University of California, San Francisco Medical Center, we have collected acoustic recordings from volunteer pneumonia patients using our prototype. At the beginning of the clinical examination, the patient was asked to sit up and the gown untied to ensure direct skin contact. Before recording began, each patient was asked to cough to clear their throat and then asked to breathe normally. For each subject, the device was placed over the manubrium, the stethoscope on the appropriate spot on the subject’s back, and the input chirp signal applied. The stethoscope was applied at six locations on the back, corresponding to the different quadrants of the lungs. A 15-s sound recording was taken at each location to allow analysis for differences in sound transmission. We obtained acoustic recordings from eight healthy subjects and five pneumonia patients, with several factors influencing inclusion in the initial study. The most important inclusion factor is that the patient had recently been diagnosed with pneumonia by chest X-ray. Healthy subjects consisted of individuals who did not have active respiratory symptoms or medical history of a confounding pulmonary pathology. The group of patients and healthy subjects were both restricted to English speakers between 18–85 years of age. The healthy group was 50% male with an average age of 27 ± 5. The patient group was 40% male with an average age of 72 ± 28.

**Figure 5:**
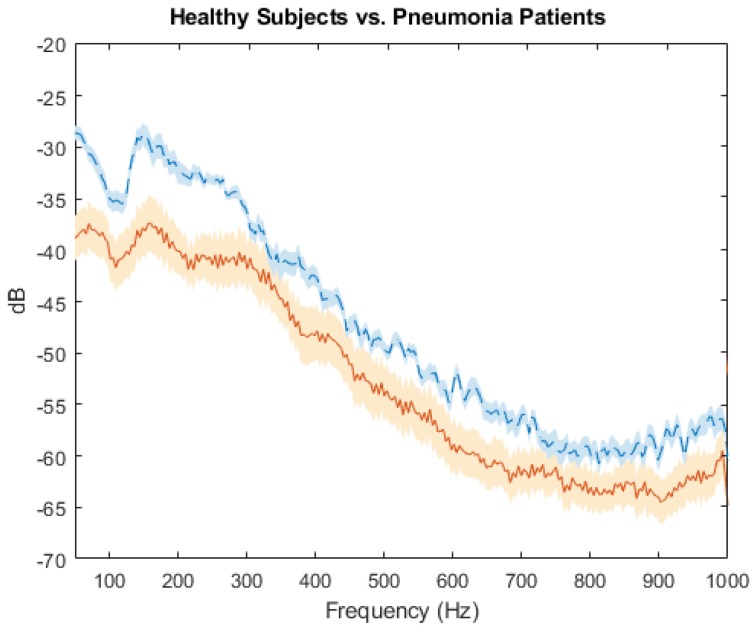
The frequency spectrum from 0–1000 Hz from the sound signal recorded from the left lower lobe of eight healthy subjects and five pneumonia patients, mean values are plotted for the pneumonia patients (solid line) and healthy subjects (dotted line) with standard error bars.

Results from the left lower lobe of five pneumonia patients and eight healthy subjects are shown in Figure 5. The average frequency response was calculated from the left lower lobe recordings of the healthy subjects and pneumonia patients known to have left lower lobe consolidation. The range of frequency for the clinical data was expanded from 500 Hz to 1000 Hz; there is an overall decrease in power for the spectrum of the pneumonia patients, compared with the healthy subjects, that is most pronounced in the range of 100 to 300 Hz. Additionally, the resonance peak occurs at 148 Hz on the spectrum for healthy subjects, compared with 160 Hz for pneumonia patients. Due to differences between thorax size and pneumonia severity, specific spectral features vary between subjects.

Although the area of consolidation was known for these patients, averaging across all lung regions may provide a more generalized method for diagnosis. In this way, a single measurement can be used to compare healthy subjects and pneumonia patients. MFCC and centroid features compress the frequency spectrum into a set of values that can be classified. The differences in spectral power result in a spectral centroid that differs between these two groups. Furthermore, the difference in shape of the spectrum can be detected through calculation of MFCC values. The MFCC and centroid values were calculated for each of the recordings to classify each subject into the healthy or pneumonia class. Using the KNN algorithm at its optimal tuning setting of K = 3, we achieved a classification accuracy of 92.3%.

## 4. Conclusions

Rapid prototyping was employed to develop a low cost, non-invasive acoustic device that uses frequency analysis to characterize structural changes in the lungs during pneumonia. Preliminary clinical results suggest that MFCC and spectral centroid values can be used to distinguish consolidated lung from healthy lung with 92.3% accuracy. These data suggest that it is feasible to develop a low-cost diagnostic tool that uses acoustic analysis to aid in the pulmonary physical exam. Although this classification accuracy is encouraging, there are limitations to this analysis. The recordings were collected from only 13 subjects, which prevented splitting the data into separate sets for training and testing and increased the risk of over-fitting. Despite this limitation, the high accuracy suggests that the pneumonia and healthy groups tend to cluster well. Future studies with larger data sets will employ leave-one-out cross validation to reduce the risk of over-fitting and also to explore classifiers suited to larger amounts of data, such as neural networks. From a device perspective, shortening the length of the chirp input will be explored in order to gather recordings without the noise from patient breathing (apnea) and the trade-off for lower frequency resolution for shorter recordings will be investigated. Future work will explore the effect of clinical features such as age and gender, which influence thorax size and could therefore be confounding variables that affect the transfer function. By including these data as inputs, we anticipate improved device performance. While our current focus is on the reliable diagnosis of pneumonia, the capabilities of our device could also be expanded to include detection of other pulmonary pathologies, such as pulmonary edema, chronic obstructive pulmonary disease, and atelectasis.

## Figures and Tables

**Table 1:** UNICEF design requirements.

Priority	Design Parameters
5 (high)	Ease of use, high accuracy
4	Long operational lifespan (>2 years)
3	Highly portable, low cost, reliable, safe, automated diagnosis, robust
2	Low training requirements, long battery life, low maintenance, high comfort
1 (low)	Little to no familiarity with technology required

Different design requirements were assigned a numerical weight by UNICEF to communicate the importance of each parameter as judged by literature review, research, surveys and interviews. Adapted from [3].

**Table 2:** Actuator Pugh chart: Scored from 1 (low) to 5 (high).

		Actuator Choice			
Criteria	*Weight*	Piezo	Solenoid	Speaker	Surf. Exciter
Intensity	*5*	1	5	2	4
Portability	*3*	5	1	4	4
Cost	*3*	4	3	3	3
Battery	*2*	4	2	4	4
Weighted Score		40	41	39	**49**

Weights of criteria are determined from the design standards listed in Table 1. Intensity has the highest weight due to its effect on accuracy. Scores quantify relative performance with respect to each criteria, where 1 is the lowest possible score and 5 is the highest.
